# Traditional Chinese herbal tea *Psychotria rubra* suppresses inflammatory response caused by respiratory tract infections via STAT3/IL-6/TNF

**DOI:** 10.1038/s41598-025-04452-z

**Published:** 2025-06-02

**Authors:** Wenlan Li, Zijie Zhang, Zixiao Jiang, Jayanthi Barasarathi, Ying Zhou, Sui Liu, Shiou Yih Lee, Yangyang Liu

**Affiliations:** 1 School of Pharmacy, Harbin University of Commerce, Harbin, 150076 China; 2 Hainan Provincial Key Laboratory of Resources Conservation and Development of Southern Medicine, Hainan Branch of Institute of Medicinal Plant Development, Key Laboratory of State Administration of Traditional Chinese Medicine for Agarwood Sustainable Utilization, International Joint Research Center for Quality of Traditional Chinese Medicine, Chinese Academy of Medical Sciences, Peking Union Medical College, Haikou, 570311 China; 3 Faculty of Health and Life Sciences, INTI International University, 71800 Nilai, Malaysia; 4 Faculty of Liberal Arts, Shinawatra University, Pathum Thani, 12160 Thailand; 5 Qionghai Hospital of Traditional Chinese Medicine, Qionghai, 571400 China

**Keywords:** Anti-inflammatory, Human health, Network Pharmacology, Rubiaceae, UPLC-Q-TOF-MS, Sore throat relief, Molecular medicine, Mass spectrometry

## Abstract

*Psychotria rubra* Herbal Tea (PRHT) is a traditional Chinese folk medicine used to treat in-flammation induced by respiratory infections. Its specific mechanisms of action, however, remain unclear. The study combined ultra-performance liquid chromatography coupled with quadrupole time-of-flight mass spectrometry (UPLC-Q-TOF-MS), network pharmacology, and in vitro cell experiments to systematically analyze the chemical composition and validate the biological activity of PRHT. Protein-protein interaction (PPI) network analysis and molecular docking were used to identify key targets and mechanisms. PRHT was found to contain 128 active compounds, including alkaloids, amino acids and their derivatives, and flavonoids. Key targets such as STAT3, IL-6, and TP53 were identified through PPI network analysis. PRHT exhibited significant anti-inflammatory effects by modulating the STAT3/IL-6/TNF-α signaling pathway. Experimental results demonstrated that PRHT inhibited the release of inflammatory cytokines in a dose-dependent manner. Molecular docking confirmed strong interactions between PRHT components and core targets. The study reveals that PRHT exerts anti-inflammatory effects by regulating the STAT3/IL-6/TNF-α signaling pathway, contributing to inflammation balance and tis-sue repair. These findings provide scientific evidence for the development of PRHT as a functional anti-inflammatory agent and offer new insights into treatment strategies for respiratory infections.

**Supplementary Information:**

The online version contains supplementary material available at 10.1038/s41598-025-04452-z.

## Introduction

Herbal teas and traditional Chinese medicine hold an important place in traditional medicine and are regarded as essential methods for regulating the balance of Yin and Yang in the body as well as strengthening health, particularly in the prevention and treatment of febrile diseases^1^. They are typically composed of multiple herbal plants that are combined according to an individual’s constitution and environmental factors to achieve a holistic therapeutic effect. In modern research, the active ingredients, biological mechanisms, and integration of herbal teas and traditional Chinese medicine with mod-ern medicine have become hot topics^2^. Key areas of research include the extraction of functional ingredients, clinical validation, and the development of scientifically backed herbal medicines and functional beverages, aiming to promote the integration and development of traditional and modern medicine^3^.

The Li tribe have lived in the tropical rainforests of Hainan Island in China for more than 3,000 years, making them the indigenous inhabitants of this land^4^. Through long-term medical practices in their battle against diseases, the people in the tribe have accumulated valuable experience, which they have integrated into their daily lives. One of their practices is that they drink herbal teas made from various plants to prevent and treat various illnesses. Among these plants, the leaves of *Psychotria rubra* (Lour.) Poir. is commonly used in the preparation of herbal teas to prevent and alleviate symptoms of colds, fever, and sore throat^5^. The name *P. rubra* is currently proposed as a synonym to *P. asiatica*^6^, yet based on the record in the Chinese Flora (http://www.cn-flora.ac.cn/, accessed on November 1, 2024), the two species are treated as two natural groups. The plant, *P. rubra* of Rubiaceae, is recorded for its properties in clearing heat and detoxifying, dispelling wind and dampness, reducing swelling, and expelling toxins, in ancient Chinese medicinal texts. It is used to treat conditions such as colds, diphtheria, sore throats, dysentery, bruises, undiagnosed swellings, abscesses, and venomous snake bites.

*Psychotria rubra* herbal tea (PRHT) has become deeply integrated into the daily life of the people in the Li tribe and is widely used in folk medicine. Despite its excellent anti-inflammatory effects, which make it popular for preventing and alleviating conditions such as colds, fever, and sore throat, the specific mechanisms in treating inflammation caused by respiratory infections, such as sore throat, remain unclear. Furthermore, systematic analysis of the chemical components of *P. rubra* and research on its therapeutic substance basis are still not well-explored. Therefore, in this study, we combined the use of UPLC-Q-TOF-MS for a systematic analysis of the chemical components of *P. rubra* and applied network pharmacology and other bioinformatics approaches to predict and analyze its mechanisms in treating inflammation caused by respiratory infections. In vitro cell experiments are also being conducted to validate the accuracy of the results. For example, Su et al.^7^ used non-targeted filtering analysis and computer simulation prediction strategies to identify the chemical components and in vivo metabolites of Dalitong Granules through UPLC-Q-TOF/MS/MS. Similarly, Chen et al.^8^. demonstrated that the Chinese medicinal herb Du Yi Wei (LH) inhibits extracellular matrix deposition to improve liver fibrosis, using methods like UPLC-Q-TOF-MS, network pharmacology, and molecular docking. This study will provide scientific evidence for the application of *P. rubra* in functional food research and lay the foundation for future studies.

## Materials and methods

### Experimental instruments

Ultra-high-performance liquid chromatograph (LC-30 A, Shimadzu), Mass spectrometer (TripleTOF 6600+, SCIEX), Centrifuge (5424R, Eppendorf), Constant temperature metal shaker (MU-G02-0448, Hangzhou MIO Instrument Co., Ltd.), analytical balance with a resolution of 0.0001 g (MS105DM, Mettler Toledo Instruments Co., Ltd.), centrifugal concentrator (CentriVap, LABCONCO), vortex mixer (VORTEX-5, Kyllin-Bell), ultrasonic cleaner (KQ5200E, Kunshan Instrument Co., Ltd.), Mouse Macrophages RAW 264.7 ( Shanghai Institutes for Life Sciences, Chinese Academy of Sciences).

### Experimental materials

Methanol (HPLC grade, Merck, Cat. No.: 1.06007.4008), acetonitrile (HPLC grade, Merck, Cat. No.: 1.00030.4008), formic acid (HPLC grade, Aladdin, Cat. No.: 695076-100ML), anhydrous ethanol (Xilong Chemical, China).

### Collection and screening of *P. rubra* components

#### Preparation of test samples

The *P. rubra* samples used in the experiment were collected from Hainan Province, China. Species identification was carried out by Mr. Li Rongtao, one of the senior researchers from the Hainan Branch of the Chinese Academy of Medical Sciences Institute of Medicinal Plants. Voucher specimen was prepared and deposited at the Hainan Provincial Chinese Medicine Herbarium under the collection number JJ20240915001. A total of 2 g of *P. rubra* was added into 50 mL of distilled water, which was then boiled in a water bath with reflux for 2 h. After cooling, the solution was filtered and transferred to a pre-dried, constant-weight evaporating dish. The leftover was cooled in a desiccator for 30 min and was quickly weighed. The residue was re-dissolved with a solvent to prepare a solution with a concentration of 1 mg/mL. A total of 100 µL of the solution was transferred into a 1.5 mL centrifuge tube, vortexed for 15 min, and centrifuged at 12,000 rpm at 4 °C for 3 min. The supernatant was filtered through a microporous membrane (0.22 μm pore size) and stored in an autosampler vial for subsequent LC-MS/MS analysis.

#### Chromatography-mass spectrometry collection conditions

The Waters ACQUITY UPLC HSS T3 Column (1.8 μm, 2.1 mm*100 mm) was selected, with 0.1% formic acid in water as mobile phase A and 0.1% formic acid in acetonitrile as mobile phase B. The column temperature was set to 40 °C, and the flow rate was 0.4 mL/min, with an injection volume of 4 µL. The gradient program started with 95% mobile phase A. At the fifth minute, the proportion of mobile phase A was reduced to 35%, then to 1% at the sixth minute, and maintained at 1% for 1.5 min. At the seventh minute and thirty-sixth second, the proportion of mobile phase A was restored to 95% and maintained until the end of the run at 10 min.

#### Screening of chemical components of *P. rubra*

The retention times of the chemical components detected in *P. rubra* were corrected, and peaks with a missing rate > 50% in the samples were filtered out. Metabolite identification was performed by searching and integrating public databases, prediction libraries, and the metDNA method. Finally, substances with an identification score above 0.5 and a QC sample CV value < 0.3 were extracted, followed by merging positive and negative modes (retaining the compounds with the highest qualitative level and smallest CV value). The SMILES numbers of the active compounds were retrieved using the PubChem database (https://pubchem.ncbi.nlm.nih.gov/, accessed on 1 November 2024), and preliminary screening was conducted using the SwissADME database (https://www.swissadme.ch/, accessed on 1 November 2024) with the following criteria: iLogP ≤ 5, nOHNH ≤ 5, nOH ≤ 10, OB ≥ 30%. Compounds not meeting these conditions were eliminated based on Lipinski’s five rules. Final screening was performed based on High GI absorption and Leadlikeness in Medicinal Chemistry, to confirm the effective active ingredients in *P. rubra*.

#### Prediction of active ingredient targets

The SwissTargetPrediction database (https://www.swisstargetprediction.ch/, accessed on November 1, 2024)^1^ and ChEMBL database (https://www.ebi.ac.uk/chembl/, accessed on November 1, 2024)^2^ were used to construct a target library for the components of *P. rubra*. The gene names of the target data were then standardized using the UniProt database (http://www.uniprot.org, accessed on 1 November 2024), and only human (*Homo sapiens*) target data were retained.

### Construction of the target set related to respiratory tract infection diseases

Genes related to respiratory tract infection diseases were retrieved from the GeneCards (https://www.genecards.org/, accessed on November 1, 2024) and DisGeNet (https://www.disgenet.org/, accessed on 1 November 2024) databases using “respiratory tract infection” as the keyword to search for the disease targets. Gene names were standardized using the UniProt database (https://www.uniprot.org/, accessed on November 1, 2024)^3^. To ensure the reliability of the data, targets with a high correlation value (≥ 15.0) were selected.

### PPI network construction and analysis

The gene targets regulated by *P. rubra* were intersected with the disease targets related to respiratory tract infections to identify the potential targets through which *P. rubra* exerts its effects in treating inflammation caused by respiratory infections. These potential targets were further screened to confirm the relevant targets for *P. rubra*’s therapeutic action in respiratory infection-induced inflammation. The resulting target gene set was then submitted to STRING (https://cn.string-db.org/, accessed on 1 November 2024)^4^ to construct a PPI network, with Homo sapiens selected as the target species. A high-confidence interaction score threshold of 0.9 was applied, and independent targets were removed while adjusting the structure of the remaining targets. The resulting PPI network was visualized in Cytoscape v3.10.0, and the DEGREE values of all interacting targets were calculated and ranked using the Cytoscape v3.10.0 plugin. The core targets related to diseases caused by respiratory infection-induced inflammation were then selected for further analysis.

### Gene ontology (GO) and KEGG pathway enrichment analysis

The potential targets of *P. rubra* in treating inflammation caused by respiratory infections were imported into the Metascape database (https://metascape.org/, accessed on 1 November 2024) and DAVID (https://david.ncifcrf.gov/, accessed on 1 November 2024) database for Gene Ontology (GO) and KEGG (www.kegg.jp/kegg/kegg1.html)^5–7^ pathway enrichment analyses. This could provide an abundant amount of biological information on the potential targets and further support the analysis on revealing the underlying mechanisms of action for *P. rubra* in treating inflammation.

### Molecular docking

By combining the number of targets and core genes involved, active components and core targets were selected for docking to predict and obtain the binding energy of protein-ligand interactions. The compound structures in sdf format were obtained from the PubChem database, and the pdb files of the core target structures were retrieved from the RCSB PDB database (https://www.rcsb.org/, accessed on 1 November 2024)^8^. Docking was performed using Autodock software (AutodockTool 1.5.7), and visualization was carried out with PyMOL (PyMol 2.5.0). The binding energy was used as an indicator to assess the binding activity and docking effect between the ligand and protein. A binding energy of < -1.4 kcal·mol-1 is generally considered to indicate a strong interaction between the two.

### The effect of PRHT on the release of inflammatory cytokines in LPS-induced RAW264.7 cells

The RAW264.7 mouse macrophages were divided into three groups: blank group (with blank culture medium), control group (with culture medium without the drug), and drug concentration treatment group (with different concentrations of the drug). The cell viability was measured using the CCK-8 method by determining the absorbance. An LPS-induced RAW264.7 mouse cell inflammation model was also established, with the cells divided into the following groups: blank group (3 mL culture medium), model group (3 mL of LPS with a final concentration of 1 µg/mL), and treatment group (1 mL of LPS with a final concentration of 1 µg/mL + 2 mL of four different drug concentrations (0.025, 0.05, 0.1, 0.2 mg/mL)). After 24 h, the cell culture supernatants were collected, and the levels of TNF-α, IL-1β, and IL-6 were measured.

Log-phase cells were cultured in a 37 °C, 5% CO_2_ incubator for 24 h to allow for adaptation. The levels of TNF-α, NO, IL-1β, and IL-6 in the cell culture supernatant were measured using an enzyme-linked immunosorbent competitive assay (ELISA). The Griess method was used to determine the absorbance of the reaction products and calculate the NO content. The NaNO_2_ standard solutions were diluted in fresh cell culture medium (DMEM + 10% FBS) to concentrations of 0, 1, 2, 5, 10, 20, 40, 60, and 100 µmol/L. A total of 50 µL of the standard solutions or cell supernatant were added to a 96-well plate, with three replicates for each concentration. Griess Reagents I and II were pre-warmed to room temperature and then added to the plate. Upon color change, absorbance at 540 nm was immediately measured using a microplate reader.

1 $${\text{Cell}}\,{\text{viability}}\left( \% \right) = \left[ {\left( {{\text{Drug}}\,{\text{group}}\,{\text{OD}} - {\text{Blank}}\,{\text{group}}\,{\text{OD}}} \right)/\left( {{\text{Control}}\,{\text{group}}\,{\text{OD}} - {\text{Blank}}\,{\text{group}}\,{\text{OD}}} \right)} \right]*100$$

2 $${\text{Inhibition}}\,{\text{rate}}\left( \% \right) = \left[ {\left( {{\text{Control}}\,{\text{group}}\,{\text{OD}} - {\text{Drug}}\,{\text{group}}\,{\text{OD}}} \right)/\left( {{\text{Control}}\,{\text{group}}\,{\text{OD}} - {\text{Blank}}\,{\text{group}}\,{\text{OD}}} \right)} \right]*100$$

### Statistical analysis method

The data were analyzed using appropriate statistical methods to evaluate the significance of the observed effects. Continuous variables were expressed as means ± standard deviation (SD). Group comparisons were performed using one-way analysis of variance (ANOVA), followed by Tukey’s post hoc test to identify specific differences between groups. In cases where data were not normally distributed, non-parametric tests. For molecular docking and gene expression analysis, binding energy scores and gene target interactions were assessed to determine the strength of the ligand-protein interactions. The results were considered statistically significant when the p-value was less than 0.05. To analyze the effects of PRHT on inflammatory cytokines in LPS-induced RAW264.7 cells, we performed an ANOVA with Tukey’s post hoc tests to compare cytokine release between the treatment and control groups. The significance level was set at *p* < 0.05 for all statistical tests. Data analysis was carried out using GraphPad Prism (version X) software.

## Results

### Chemical composition and confirmation of active ingredients in PRHT

**Fig. 1:**
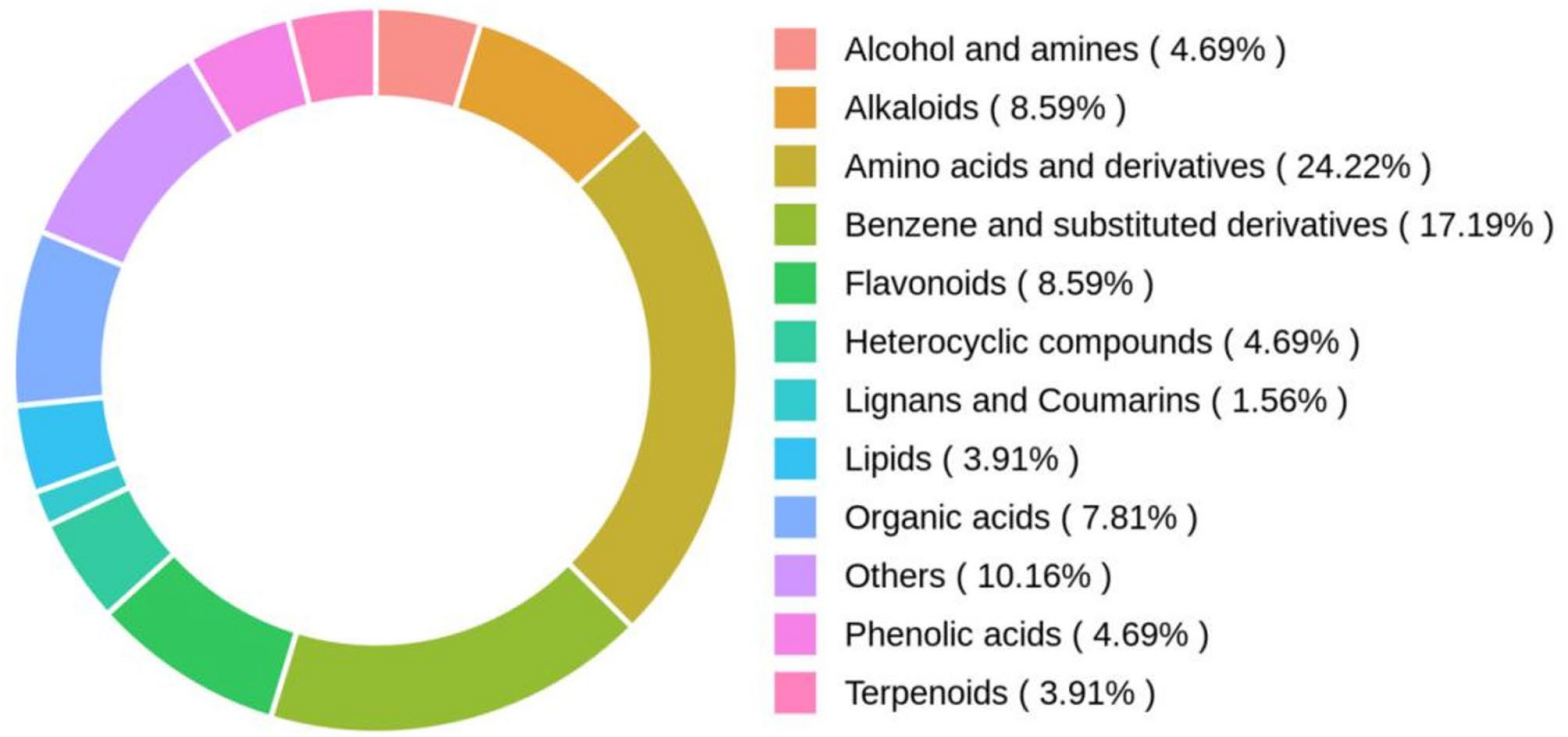
Composition of chemical components of *P. rubra.*

Through UHPLC-Q-TOF-MS untargeted metabolomics analysis, a total of 128 active components with high gastrointestinal absorption rates were identified under the conditions of iLogP ≤ 5, nOHNH ≤ 5, nOH ≤ 10, and OB ≥ 30%, combined with Lipinski’s five rules and High GI absorption (Fig. 1). These components include: 31 amino acids and derivatives, 22 benzenes and substituted derivatives, 11 alkaloids, 11 flavonoids, 10 organic acids, six alcohols and amines, six heterocyclic compounds, five lipids, five phenolic acids, six terpenoids, two lignans and coumarins, and 13 other components.

Using “respiratory tract infection” as the search term, relevant disease genes were screened based on their compatibility, and genes related to respiratory tract infections were integrated from multiple databases. A total of 943 unique gene targets were identified. Among the 128 active components, 120 were found to intersect with the respiratory tract infection-related disease gene set. These 120 active components shared a total of 207 unique overlapping targets with respiratory tract infection-related diseases.

### PPI network construction and analysis

**Fig. 2:**
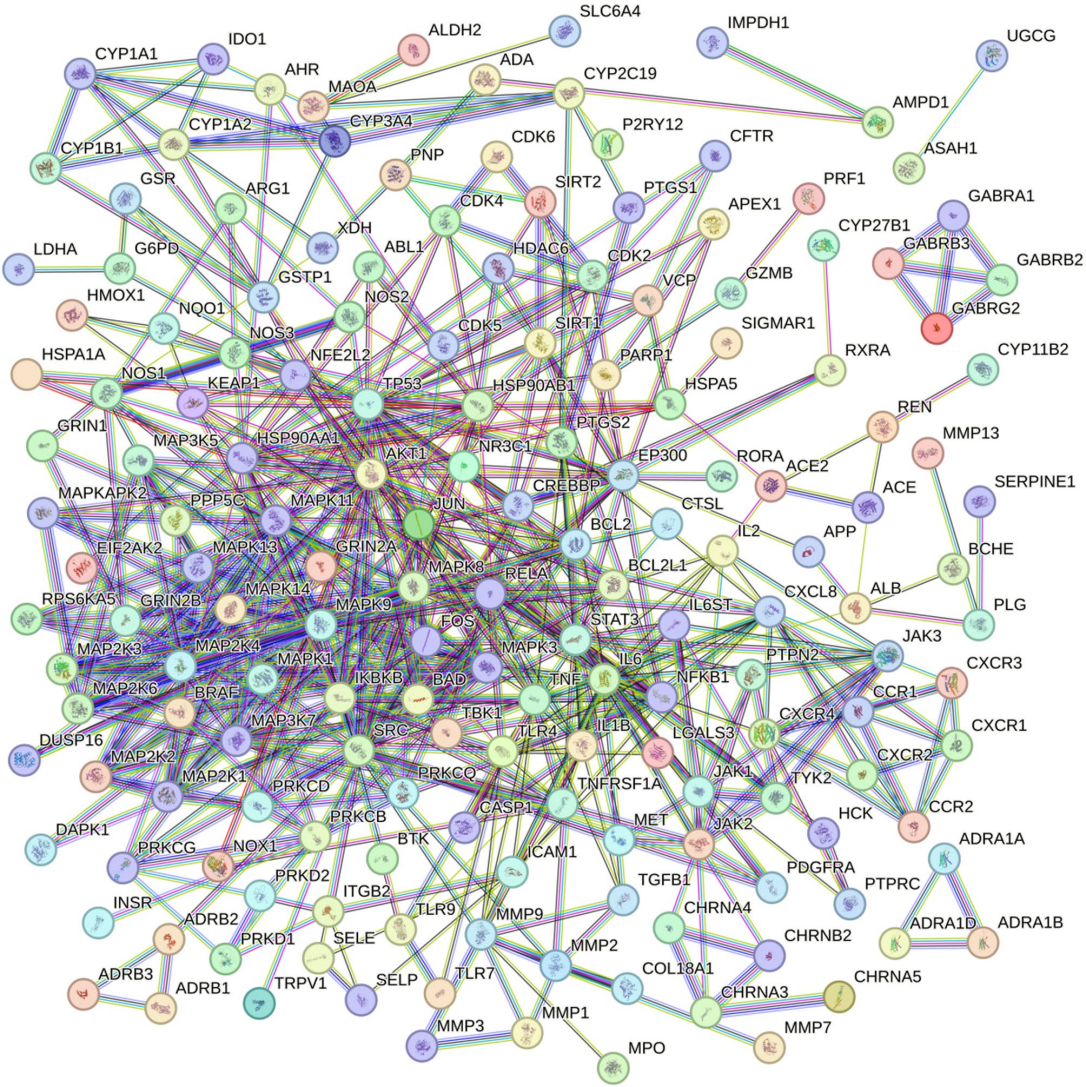
Protein-protein interaction network diagram.

Based on the STRING protein-protein interaction background network, a PPI network was constructed for the 120 active components of *P. rubra* in the treatment of respiratory tract infection-related diseases, with a confidence threshold of 0.9 to optimize the network construction. In the PPI network of *P. rubra* in treating respiratory infections, there were 207 nodes and 593 edges, with an average node degree of 5.73 and an average clustering coefficient of 0.47 (Fig. 2).

Using four algorithms-EPC, MCC, MNC, and DEGREE (with DEGREE results as the primary outcome)-the top 10 core targets with the highest centrality in the treatment of respiratory tract infection-related diseases were identified (Table 1). These targets are: TP53 (31), STAT3 (29), JUN (27), MAPK8 (26), MAPK1 (26), AKT1 (26), SRC (26), MAPK3 (25), IL6 (23), and MAPK9 (23).

**Table 1:** Data related to core target screening.

Name	EPC	MCC	MNC	DEGREE
TP53	39.05	0.36	29	31
STAT3	37.75	0.37	28	29
JUN	39.52	0.45	27	27
MAPK8	39.26	0.41	26	26
MAPK1	38.59	0.39	26	26
AKT1	37.68	0.34	25	26
SRC	36.52	0.31	21	26
MAPK3	38.08	0.38	25	25
IL6	34.88	0.35	23	24
MAPK9	38.10	0.44	23	23

### Gene ontology (GO) functional enrichment

**Fig. 3:**
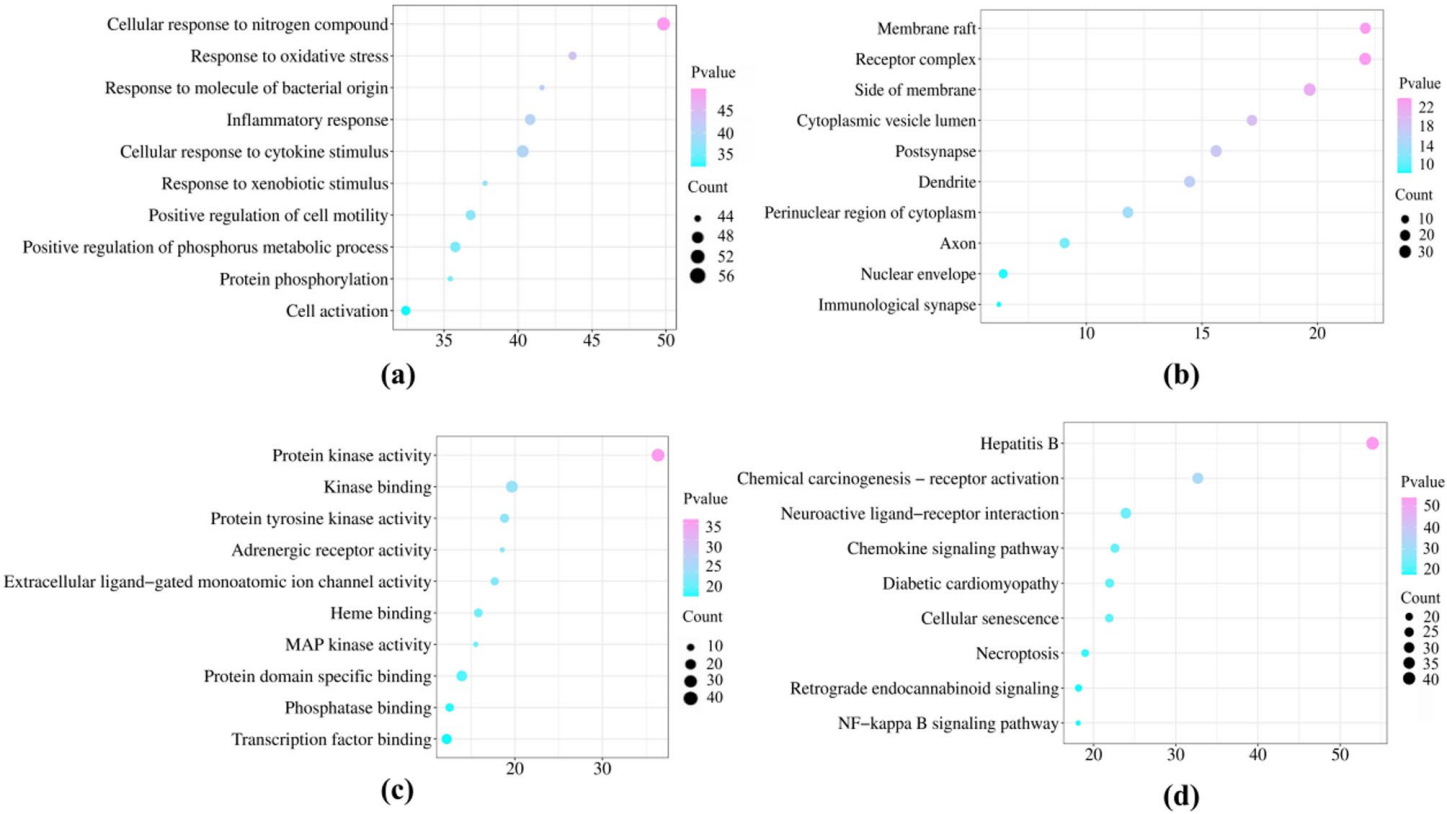
Gene Ontology (GO) functional enrichment and KEGG pathway enrichment analysis. (**a**) GO biological processes; (**b**) GO cellular components; (**c**) GO molecular functions; (**d**) KEGG pathway.

A potential biological function analysis was conducted for the 120 active component targets of *P. rubra*. The top 10 items in each category of GO enrichment were visualized based on the P-values. In the molecular function analysis, *P. rubra* was mainly involved in protein kinase activity (*p* = 4.17*10⁻³⁷), kinase binding (*p* = 2.19*10⁻²⁰), protein tyrosine kinase activity (*p* = 1.51*10 − 1⁹), adrenergic receptor activity (*p* = 2.69*10 − 1⁹), extracellular ligand-gated monoatomic ion channel activity (*p* = 2.00*10 − 1⁸), and others. In terms of biological processes, the active components of *P. rubra* were primarily involved in cellular response to nitrogen compound (*p* = 1.45*10⁻⁵⁰), response to oxidative stress (*p* = 2.04*10⁻⁴⁴), response to molecule of bacterial origin (*p* = 2.40*10⁻⁴²), inflammatory response (*p* = 1.51*10⁻⁴¹), cellular response to cytokine stimulus (*p* = 4.90*10⁻⁴¹), and more. For cellular components, *P. rubra* was affecting mainly the membrane raft (*p* = 8.32*10⁻²³), receptor complex (*p* = 8.51*10⁻²³), side of membrane (*p* = 2.14*10⁻²⁰), cytoplasmic vesicle lumen (*p* = 6.76*10 − 1⁸), postsynapse (*p* = 2.40*10 − 1⁶), and others (Fig. 3).

### Construction of the “active ingredients-efficacy targets-action pathways” network diagram of PRHT

**Fig. 4:**
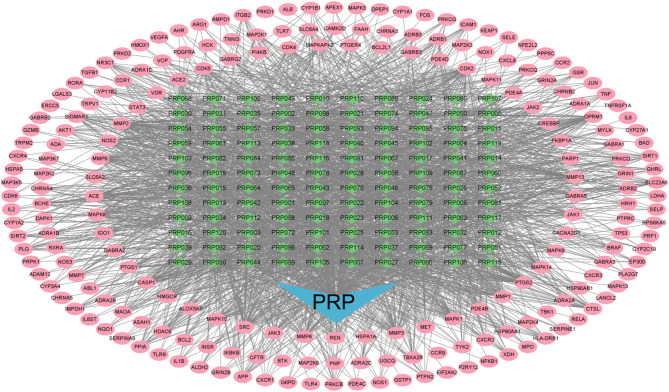
Network diagram of “active ingredient - efficacy target - pathway of action”.

Based on the active components of *P. rubra* and their efficacy targets, combined with the gene targets related to respiratory tract infections, the “active components-efficacy targets-action pathways” network diagram for *P. rubra* was constructed using Cytoscape 3.10.0 software. The corresponding action targets for each active component in *P. rubra* were revealed (Fig. 4). In the network, the pink oval nodes (207) represent the disease targets involved in respiratory tract infections, while green diamond-shaped nodes (120) represent the active components of *P. rubra* that play a role in treating respiratory tract infection symptoms. The lines connecting the components and targets indicate that the target can be regulated by the corresponding component.

### Molecular docking

**Fig. 5:**
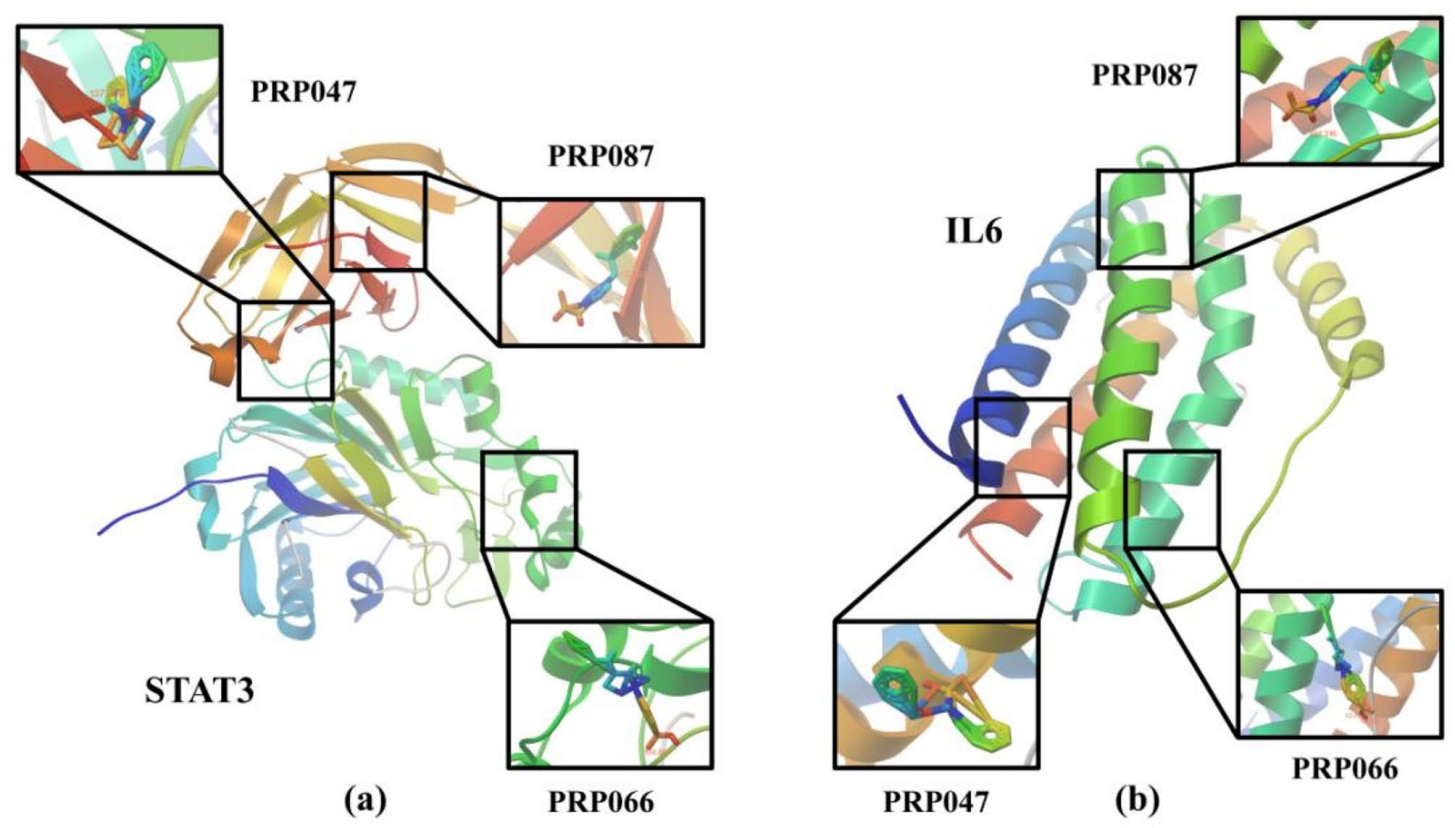
Schematic of molecular docking of *P. rubra* (Lour.) Poir. (**a**) STAT3 with two compounds; (**b**) IL6 with two compounds.

By combining the number of targets and core genes, STAT3 and IL6 were selected as the docking center targets, and N-phenylacetylphenylalanine (PRP047), carboxytolbutamide (PRP066), and acetaminosalol (PRP087) were chosen as the core components (Fig. 5). The molecular docking results showed that the binding energies of STAT3 and IL6 with the three active components were all less than − 1.4 kcal·mol^− 1^, indicating that the central target genes have a strong binding affinity with these three components (Table 2). Specifically, the binding energy of PRP047 with STAT3 was − 7.02 Kcal·mo^− 1^ and with IL6 was − 4.60 Kcal·mol^− 1^; the binding energy of PRP066 with STAT3 was − 7.45 Kcal·mol^− 1^ and with IL6 was − 6.23 Kcal·mol^− 1^; the binding energy of PRP087 with STAT3 was − 6.82 Kcal·mol^− 1^ and with IL6 was − 5.46 Kcal·mol^− 1^. Additionally, we predicted the ADMET of the three active components (Table 3), and the results indicated that these three active substances all meet Lipinski’s rule. This further suggests that the active components of P. rubra can effectively interact and bind with the core targets.

**Table 2:** Active ingredient and core target Docking information.

Gene	Active ingredient	Binding energy Kcal·mol-1	Gene	Active ingredient	Binding energy Kcal·mol-1
STAT3	PRP047	-7.02	IL6	PRP047	-4.60
	PRP066	-7.45		PRP066	-6.23
	PRP087	-6.82		PRP087	-5.46

**Table 3:** ADMET prediction of compound PRP047, PRP066 and PRP087.

Compound	mol /MW	Log *P*_o/w_ (iLOGP)	donorHB	accptHB	Bioavailability Score
N-phenylacetylphenylalanine (PRP047)	283.32	1.91	2	3	0.85
Carboxytolbutamide (PRP066)	300.33	1.18	3	5	0.56
Acetaminosalol (PRP087)	271.27	1.85	2	4	0.55

### The effect of PRHT on the release of inflammatory cytokines in LPS-induced RAW264.7 cells

**Fig. 6:**
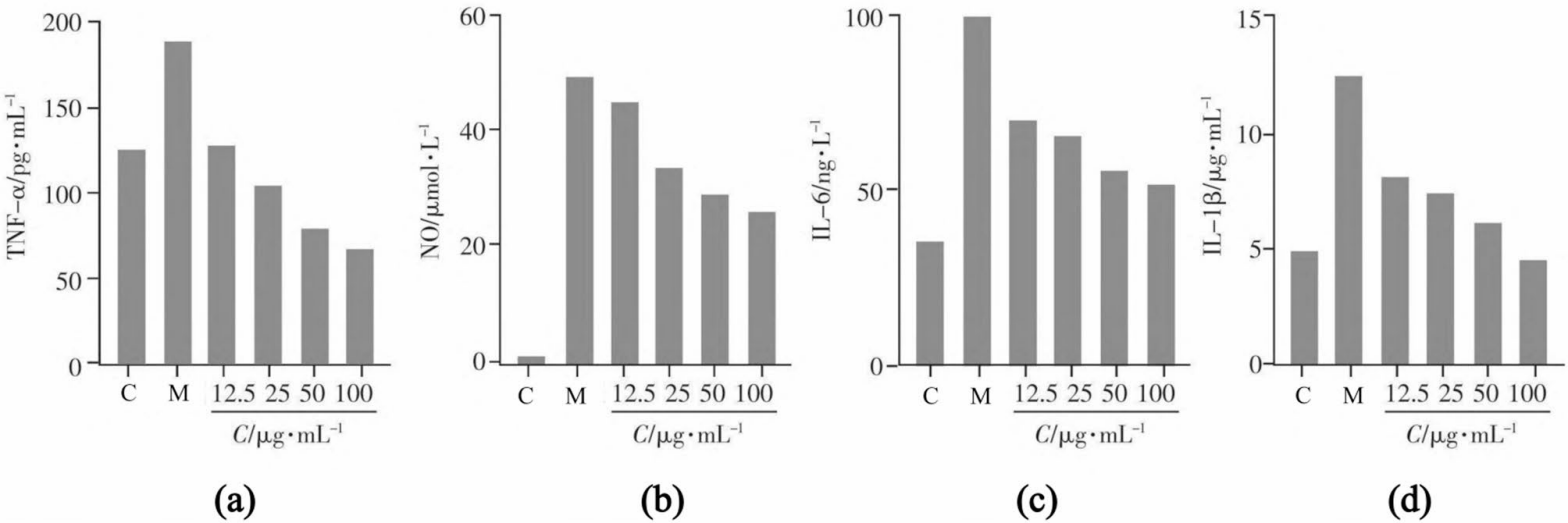
Inhibitory effects of iridin on (**a**) TNF-α, (**b**) NO, (**c**) IL-1β, and (**d**) IL-6 production by LPS - activated RAW 264.7 cells (*n* = 3).

In the normal group, due to the cells’ inherent defense mechanisms, a certain number of inflammatory mediators were still being produced. Compared to the control group, the levels of the four inflammatory mediators released by macrophages in the model group significantly increased after being stimulated with 1 µg/mL lipopolysaccharide (LPS) for 24 h, confirming the establishment of a successful model. In comparison to the model group, significant differences (*P* < 0.001) were observed in the inhibition of TNF-α, NO, IL-1β, and IL-6 release in macrophages treated with 25–200 µg/mL of the drug, showing a dose-dependent response (Fig. 6). At a concentration of 100 µg/mL, the levels of inflammatory factors (TNF-α, IL-1β, IL-6) in the cells were lower than those of the normal group, suggesting that PRHT, under external stimulation (LPS induction), may enhance the phagocytic ability of RAW 264.7 cells and improve the body’s defense against exogenous substances.

## Discussion

### The mechanism of action and innovation in the interaction between PRHT metabolites and molecular targets

The metabolites identified in this study, along with the predicted molecular targets, support the traditional use of *P. rubra* in alleviating throat pain. By targeting and regulating the STAT3, IL-6, and TNF-α signaling pathways, PRHT demonstrates significant anti-inflammatory effects, validating its traditional functions of clearing heat, detoxifying, reducing swelling, and relieving pain. PRHT acted on 168 protein targets in the treatment of inflammatory responses caused by respiratory tract infections. These target genes have distinct functions. For example, TP53 is a transcription factor that regulates target gene expressions to induce various functions, including cell cycle arrest, DNA repair, apoptosis, senescence, and inhibition of angiogenesis^9,10^. The P53 protein encoded by TP53 can regulate cell division and proliferation^11^. STAT3, located on chromosome 17, responds to cytokines and growth factors, and is phosphorylated by receptor-associated kinases to form homodimers or heterodimers. These dimers translocate to the cell nucleus, acting as transcriptional activators. STAT3 is activated by phosphorylation in response to various cytokines and growth factors (including IFN, EGF, IL5, IL6, HGF, LIF, and BMP2) and mediates the expression of multiple genes, playing a crucial role in various cellular processes such as cell growth and apoptosis^12,13^. JUN, located on chromosome 1, directly interacts with specific DNA sequences to regulate gene expression^14,15^. MAPK1, MAPK3, and MAPK8 are members of the MAP kinase family, involved in various cellular processes such as cell proliferation, differentiation, transcriptional regulation, and development. MAP kinases serve as integration points for multiple biochemical signals, activated by various cellular stimuli, and regulate immediate early gene expression in response to cellular stimulation through specific transcription factors^16,17^. IL-6 is a cytokine with multiple biological functions, serving as a potent inducer of the acute-phase response. It plays an important role in the final differentiation of B cells into Ig-secreting cells, acting on B cells, T cells, hepatocytes, hematopoietic cells, and cells in the central nervous system. It can also act as actin, released into the bloodstream after muscle contraction, promoting fat breakdown^18^. SRC (proto-oncogene tyrosine-protein kinase Src) is a non-receptor protein tyrosine kinase, activated after interaction with many different types of cell receptors (including immune response receptors, integrins, other adhesion receptors, receptor protein tyrosine kinases, G protein-coupled receptors, and cytokine receptors). SRC is involved in regulating multiple signaling pathways that control various biological activities, including gene transcription, immune response, cell adhesion, cell cycle progression, apoptosis, migration, and transformation^19^.

Moreover, compared to other studies on herbal teas and traditional medicine formulas, this study is the first to comprehensively analyze the active ingredients of PRHT by integrating untargeted metabolomics and network pharmacology, and to verify its biological activity through in vitro experiments, showcasing the innovative nature of interdisciplinary integration. The findings not only deepen the understanding of the molecular mechanisms of PRHT but also provide scientific evidence for its development as a functional health beverage, highlighting its important application potential.

### The synergistic effects and anti-inflammatory mechanism of PRHT metabolites

The network pharmacology analysis in this study suggests that the active components of *P. rubra* synergistically act on STAT3 and IL-6, regulating the balance between pro-inflammatory and anti-inflammatory factors. During the initiation phase of inflammation, the pro-inflammatory actions of TNF-α and IL-6 dominate, helping the body clear pathogens and recruit immune cells^20^. The active small molecules in *P. rubra* herbal tea act on IL-6 to activate STAT3, which begins to limit excessive inflammation. STAT3 inhibits the overexpression of TNF-α by downregulating NF-κB activity.

During the resolution phase of inflammation, IL-6 and TNF-α enhance the anti-inflammatory function of STAT3, accelerating tissue repair and immune tolerance. IL-6 and TNF-α work together to promote the formation of M2 macrophages, while STAT3 further regulates the secretion of anti-inflammatory factors from M2 macrophages, maintaining the stability of the immune system. IL-6-activated STAT3 induces the expression of SOCS3, which inhibits the pro-inflammatory actions of IL-6 and TNF-α, forming a self-limiting negative feedback loop. This feedback mechanism prevents prolonged inflammation and helps sustain an anti-inflammatory state^20^.

**Fig. 7:**
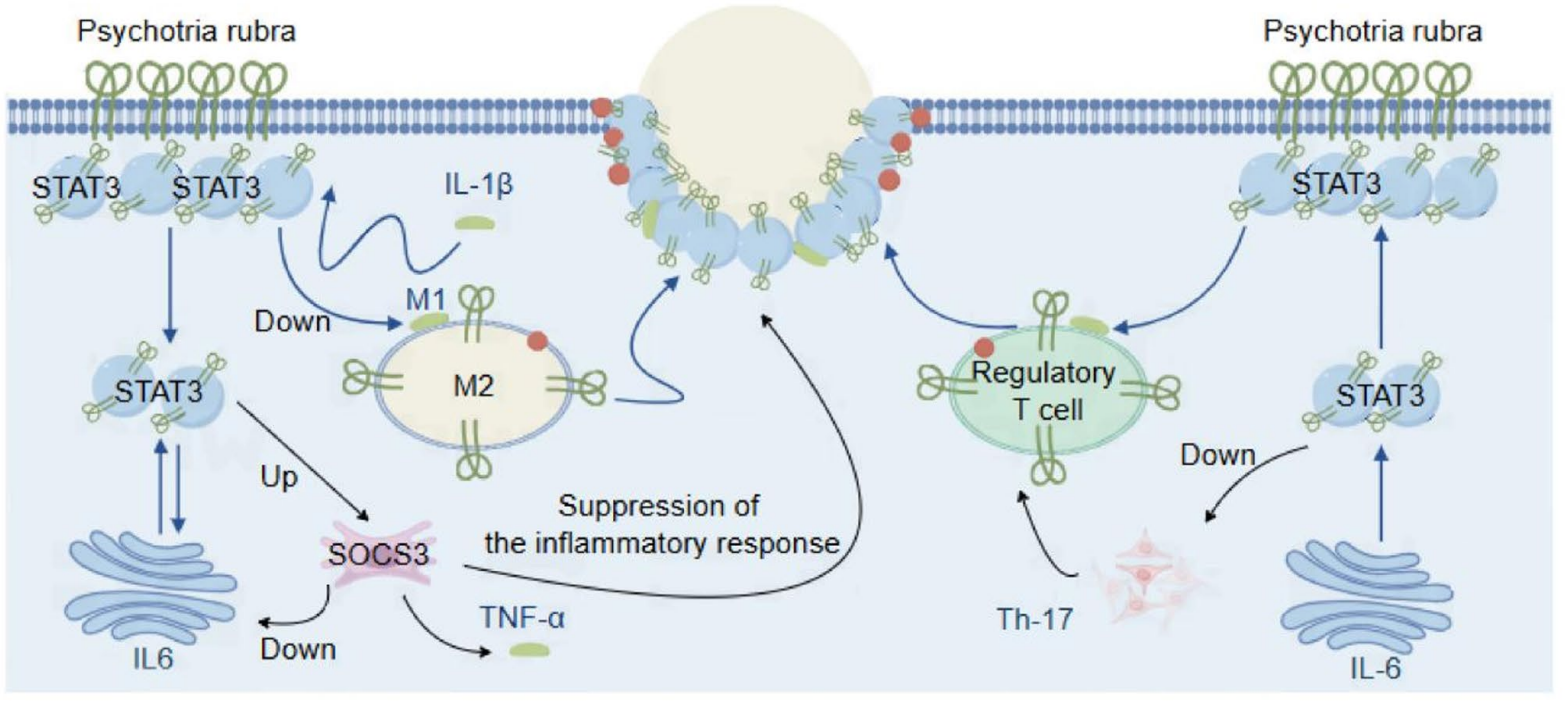
The synergistic effects and anti-inflammatory mechanism of PRHT metabolites.

In summary, in the treatment of inflammation caused by respiratory tract infections, the active components of PRHT play a key role through their synergistic actions with STAT3, TNF-α, and IL-6 (Fig. 7). They dynamically regulate the inflammatory response through complex signaling networks, demonstrating high coordination and multifunctionality in maintaining inflammation balance, preventing tissue damage, and promoting repair.

### Limitations of the study and future directions

The primary limitation of this study lies in the absence of in vivo experimental validation and clinical trial support, which limits the immediate applicability of the findings. However, we would like to clarify our research strategy. This study focuses on systematically analyzing the chemical components of *Psychotria rubra* and exploring their potential mechanisms using UPLC-Q-TOF-MS and network pharmacology approaches. The goal is to establish a theoretical foundation and provide preliminary data for future in-depth studies. We believe that in vitro experiments and chemical composition analysis are essential initial steps in understanding the therapeutic potential of this herbal tea, while animal studies and clinical trials will serve as subsequent validation stages based on more comprehensive data.

It is also important to note that animal experiments and clinical trials involve more complex ethical approvals and resource demands. To ensure the scientific rigor and reliability of our data, we plan to carry out animal studies after further verifying our initial findings. Therefore, we have adopted a phased research strategy to gradually incorporate animal experiments in future studies, allowing us to validate and expand upon our preliminary discoveries more effectively.

In addition, other potential mechanisms that have not yet been fully explored, such as the NF-κB and MAPK signaling pathways, should also be considered in future work. The NF-κB pathway plays a crucial role as both an initiator and a key regulator in the inflammatory process. It can be activated by pro-inflammatory cytokines such as IL-6, TNF-α, and IL-1, which stimulate the IκB kinase (IKK) complex^21^. This activation leads to the phosphorylation and degradation of IκB (an NF-κB inhibitor), thereby releasing the p65/p50 NF-κB dimer to translocate into the nucleus and initiate the expression of inflammatory genes.

The MAPK signaling pathway, a widely distributed intracellular signaling cascade, regulates cell growth, differentiation, and stress responses. It comprises three core branches: ERK, p38, and JNK, each of which plays dual roles in the regulation of inflammation. In inflammatory diseases such as sepsis, pneumonia, and atherosclerosis, the bidirectional regulatory effects of MAPK on inflammatory gene expression present potential therapeutic targets. Suppressing the abnormal activation of p38 or JNK, or activating the ERK pathway, can mitigate inflammatory damage and exert anti-inflammatory effects^22,23^.

In summary, as conditions permit, we intend to progressively introduce animal experiments and randomized controlled clinical trials to further explore the effects of PRHT in various inflammation models and uncover additional pharmacological pathways. Given that PRHT is considered an herbal beverage, optimizing its formulation may further support its development into a functional drink suitable for a broad population.

## Conclusions

This study systematically analyzed the chemical components of PRHT, and through network pharmacology and cell experiments, we successfully revealed the underlying molecular mechanisms on its anti-inflammatory effects. It has been proven that the active ingredients of PRHT exert significant anti-inflammatory effects by regulating the STAT3, IL-6, and TNF-αsignaling pathways. Key active components, including N-phenylacetylphenylalanine, carboxytolbutamide, and acetaminosalol, were shown to bind well to the core targets STAT3 and IL-6 in molecular docking, confirming their traditional use in alleviating inflammation-related symptoms such as sore throat. The metabolites of PRHT not only demonstrated synergistic effects in multi-target regulation but also exhibited multifunctionality in promoting inflammation balance and tissue repair.

These findings provide strong scientific evidence for PRHT as a promising natural anti-inflammatory agent, supporting its potential use as a functional food ingredient. Future developments on this valuable herbal tea could benefit the local health beverage sector, as well as offering a new approach in anti-inflammatory health management.

## Electronic supplementary material

Below is the link to the electronic supplementary material.

Supplementary Material 1

## Data Availability

All data generated or analysed during this study are included in this published article and its supplementary information files.
