# Supplementary material for: Traditional Chinese herbal tea Psychotria rubra suppresses inflammatory response caused by respiratory tract infections via STAT3/IL-6/TNF
Source: Sci Rep. 2025 Jun 2;15:19325. doi: 10.1038/s41598-025-04452-z (PMC12130517; doi:10.1038/s41598-025-04452-z)
Supplement: Supplementary file 1 — Supplementary Material 1 [file 41598_2025_4452_MOESM1_ESM.docx]

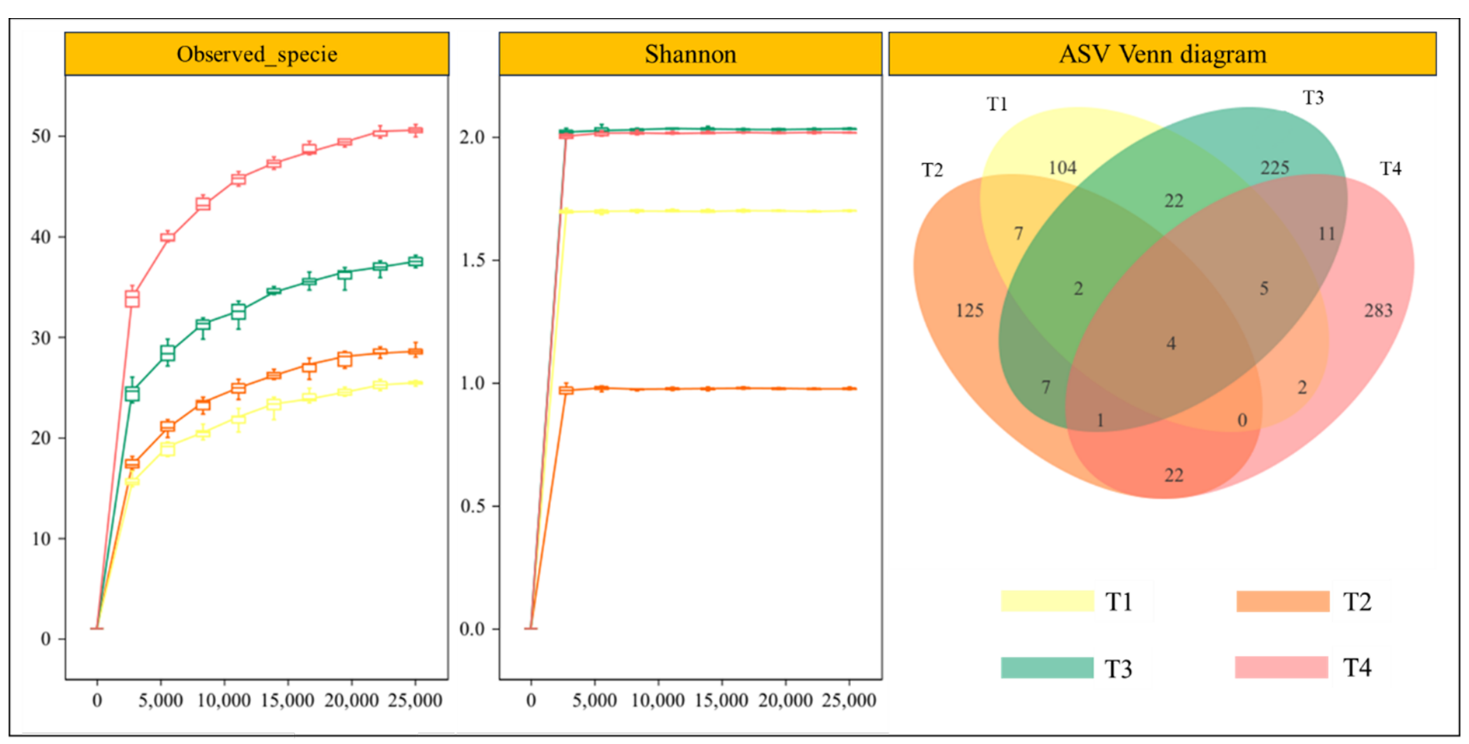


**Figure S1.** Dilution curves of the ITS1 region sequences of the endophytic fungi of the sample plants and the Wayne diagram of the ASV of the endophytic fungi of the sample plants.

**Table1 S1.** Statistics of sequences from the two halophytes after filtering out low-quality and chimeric reads based on DADA2.

| **SampleID** | **Input** | **Filtered** | **Denoised** | **Merged** | **Non-chimeric** | **Non-singleton** |
| --- | --- | --- | --- | --- | --- | --- |
| T1_1_g | 78178 | 66670 | 66613 | 65660 | 62013 | 62013 |
| T1_1_j | 64898 | 61536 | 61510 | 61168 | 61045 | 61045 |
| T1_1_y | 97423 | 90629 | 90504 | 89236 | 76743 | 76742 |
| T1_2_g | 75532 | 63640 | 63613 | 62337 | 59643 | 59643 |
| T1_2_j | 82094 | 77019 | 76996 | 76489 | 75982 | 75982 |
| T1_2_y | 80769 | 74821 | 74785 | 64830 | 63185 | 63185 |
| T1_3_g | 70512 | 65036 | 65017 | 64833 | 63898 | 63898 |
| T1_3_j | 69420 | 65271 | 65194 | 64747 | 60997 | 60997 |
| T1_3_y | 83277 | 77777 | 77753 | 77654 | 73297 | 73297 |
| T2_1_g | 120055 | 101982 | 101963 | 101399 | 96718 | 96718 |
| T2_1_j | 90594 | 72640 | 72471 | 71702 | 65531 | 65531 |
| T2_1_y | 108125 | 81418 | 81387 | 80747 | 79231 | 79231 |
| T2_2_g | 209375 | 109773 | 109689 | 109192 | 107737 | 107737 |
| T2_2_j | 93052 | 69635 | 69584 | 69445 | 63396 | 63395 |
| T2_2_y | 94578 | 60311 | 60255 | 59873 | 58742 | 58742 |
| T2_3_g | 94474 | 87547 | 87471 | 87104 | 84726 | 84725 |
| T2_3_j | 91748 | 72336 | 72150 | 71705 | 71705 | 71705 |
| T2_3_y | 93594 | 50994 | 50960 | 50581 | 50575 | 50575 |
| T3_1_g | 359573 | 158343 | 158084 | 64381 | 63725 | 63721 |
| T3_1_j | 101098 | 91951 | 91771 | 90098 | 88566 | 88566 |
| T3_1_y | 114101 | 107583 | 107492 | 106430 | 93804 | 93803 |
| T3_2_g | 86937 | 71022 | 70991 | 70591 | 66128 | 66128 |
| T3_2_j | 99360 | 94288 | 94111 | 92869 | 81426 | 81426 |
| T3_2_y | 92271 | 85106 | 85053 | 84782 | 80408 | 80408 |
| T3_3_g | 83144 | 71984 | 71970 | 71551 | 67590 | 67590 |
| T3_3_j | 90844 | 86322 | 86068 | 85520 | 79161 | 79161 |
| T3_3_y | 102285 | 95058 | 95026 | 94355 | 91621 | 91621 |
| T4_1_g | 89250 | 83305 | 83260 | 83067 | 82183 | 82183 |
| T4_1_j | 324673 | 32766 | 32316 | 29966 | 26382 | 26382 |
| T4_1_y | 335860 | 75085 | 74422 | 70964 | 61511 | 61511 |
| T4_2_g | 95620 | 86891 | 86746 | 86673 | 86266 | 86266 |
| T4_2_j | 84525 | 77880 | 77829 | 77689 | 76667 | 76667 |
| T4_2_y | 101972 | 94873 | 94821 | 94340 | 94160 | 94160 |
| T4_4_g | 90403 | 81353 | 81216 | 80857 | 80339 | 80339 |
| T4_4_j | 92250 | 86541 | 86415 | 85929 | 83832 | 83832 |
| T4_4_y | 111172 | 103528 | 103458 | 103312 | 100008 | 100008 |

**Table S2.** Codes of bioclimatic variables according to WorldClim database (version 2.0, http://worldclim.org/version2, accessed 10 November 2021).

| Code | Bioclimatic Variable |
| --- | --- |
| BIO1 | Annual mean temperature (°C) |
| BIO2 | Mean diurnal range (mean of monthly, maximum  temperature—minimum temperature) (°C) |
| BIO3 | BIO3 Isothermality (BIO2/NIO7) ($\times$100) |
| BIO4 | Temperature seasonality (standard deviation _100) |
| BIO5 | Maximum temperature of warmest month (°C) |
| BIO6 | Minimum temperature of coldest month (°C) |
| BIO7 | Temperature annual range (BIO5-BIO6) (°C) |
| BIO8 | Mean temperature of wettest quarter (°C) |
| BIO9 | Mean temperature of driest quarter (°C) |
| BIO10 | Mean temperature of warmest quarter (°C) |
| BIO11 | Mean temperature of coldest quarter (°C) |
| BIO12 | Annual precipitation (mm) |
| BIO13 | Precipitation of wettest month (mm) |
| BIO14 | Precipitation of driest month(mm) |
| BIO15 | Precipitation seasonality (coefficient of variation) (mm) |
| BIO16 | Precipitation of wettest quarter (mm) |
| BIO17 | Precipitation of driest quarter (mm) |
| BIO18 | Precipitation of warmest quarter (mm) |
| BIO19 | Precipitation of coldest quarter (mm) |
